# What Justifies Judgments of Inauthenticity?

**DOI:** 10.1007/s10730-018-9356-5

**Published:** 2018-07-03

**Authors:** Jesper Ahlin

**Affiliations:** Division of Philosophy, KTH Royal Institute of Technology, Stockholm, Sweden

**Keywords:** Authenticity, Autonomy, Decision-making, Paternalism, Bioethics

## Abstract

The notion of authenticity, i.e., being “genuine,” “real,” or “true to oneself,” is sometimes held as critical to a person’s autonomy, so that inauthenticity prevents the person from making autonomous decisions or leading an autonomous life. It has been pointed out that authenticity is difficult to observe in others. Therefore, judgments of inauthenticity have been found inadequate to underpin paternalistic interventions, among other things. This article delineates what justifies judgments of inauthenticity. It is argued that for persons who wish to live according to the prevailing social and moral standards and desires that are seriously undesirable according to those standards, it is justified to judge that a desire is inauthentic to the extent that it is due to causal factors that are alien to the person and to the extent that it deviates from the person’s practical identity. The article contributes to a tradition of thinking about authenticity which is known mainly from Frankfurt and Dworkin, and bridges the gap between theoretical ideals of authenticity and real authenticity-related problems in practical biomedical settings.

## Introduction

Personal autonomy, i.e., self-determination, is a central notion in contemporary bioethics. Generally speaking, a person is autonomous if she is self-governed. Factors that undermine autonomy include, for instance, lacking decision-making capacities and controlling influences such as coercion or manipulation. Sometimes authenticity, i.e., being “genuine,” “real,” “true to oneself,” or similar, is held as critical to a person’s autonomy, so that inauthenticity prevents the person from making autonomous decisions or leading an autonomous life. It is a bioethical problem how authenticity should be understood. Various theories have been proposed with the intention of conceptualizing authenticity; none takes complete precedence over others.

In practice, the notion is relevant mainly in considerations of what justifies judgments of inauthenticity. For instance, patients suffering from borderline personality disorder (BPD) sometimes display sudden and dramatic shifts in goals, values, vocational aspirations, choice of friends, and so on (Lester 2009, p. 284). During a short time span, a BPD patient can both request medication, as only that enables her to go through psychotherapy, and refuse medication, as one of its side effects is that it clouds her thinking. Healthcare personnel cannot adhere to both wishes. Caretakers with autonomy-promoting or paternalistic ambitions may be interested in whether it is justified to treat any of the BPD patient’s decisions as inauthentic, and if so, on what grounds. Other examples include late stage schizophrenics who are completely indifferent to how their lives go (cf. American Psychiatric Association 2013) and anorectics who report that they would rather die than gain weight (Tan et al. 2006). In such cases, it is sometimes relevant to ask whether it is justified to treat patients’ wishes as inauthentic.

Thus, there is a three-step problem regarding the notion of authenticity. First, it is unclear under which conditions something or someone is authentic or inauthentic. Second, it is difficult to know whether something or someone meets those conditions. Mainly for reasons of epistemic uncertainty, it is therefore unclear what justifies the judgment that something or someone is inauthentic. Third, some paternalistic or authenticity-promoting interventions may also be justified in light of judgments of inauthenticity. This article is only concerned with the second step of this three-step problem.

I argue that for persons who wish to live according to the prevailing social and moral standards and desires that are seriously undesirable according to those standards, it is justified to judge that a desire is inauthentic to the extent that it is due to causal factors that are alien to the person and to the extent that it deviates from the person’s practical identity. My arguments in this article contribute to a tradition of thinking about authenticity which is mainly known from Frankfurt (1971) and Dworkin (1988) and has recently been supported by Juth (2005) and DeGrazia (2005), among others. However, my contribution is more practical than the theoretical ideals proposed by those authors; this article is an attempt to bridge the gap between theoretical ideals of authenticity and real authenticity-related problems in practical biomedical settings.

The article has two main sections and is structured as follows. In the first main section, I elaborate on how the notion of authenticity is relevant to biomedicine and introduce two recent attempts to collect theories of authenticity in taxonomies. I spell out my proposal of what justifies judgments of inauthenticity in the second main section. My proposal builds on the arguments in the first section, which is why the first section is rather detailed and takes up much space. A brief final section concludes.

## Theorizing About Authenticity

### Authenticity and Biomedicine

Autonomy is one of the main guiding principles in contemporary bioethics. In the standard model of autonomy, a person is autonomous with respect to her desires or actions to the extent that they are due to her own self, and not due to some other influencing force, be it internal or external to her (cf. Taylor 2005a). Bioethicists usually invoke two main notions with regard to patients’ autonomy; decision-making capacity and voluntariness. That is, if a patient is competent to make healthcare decisions, according to, e.g., MacCAT-T standards of decisional-capacity (Grisso et al. 1997), and does so without undue influences internal or external to the patient (Nelson et al. 2011), most bioethicists agree that the patient’s decisions should be respected. Sometimes a third notion is raised, namely that of authenticity. The reasons vary. A patient may make healthcare decisions that seem to be “out of character,” or that seem to conform to others’ wishes rather than to her own, or she may suffer from some medical disorder that seems to affect her values. Accordingly, the notion of authenticity has increasingly gained theorists’ attention (see, e.g., Bauer 2017; Sjöstrand and Juth 2014; White 2017).

It has become clear that there is no consensus regarding how authenticity should be understood, or what exactly it may add to the concern for patients’ autonomy. To the contrary, various theoretical approaches are present in bioethical conversations, none of which takes precedence over others and, arguably, none of which manages to solve all authenticity-related problems that bioethicists have raised. Below, I account for two attempts to collect theories of authenticity in taxonomies that enable overview and analysis. These accounts will be relevant to the arguments in the subsequent section. However, before proceeding, some delimitations are necessary.

The notion of authenticity has been understood to apply to different things. Some argue that it is the authenticity of persons that is of importance to autonomy theory (cf. Bauer 2017). Others hold that it is the authenticity of a person’s life that should be considered (cf. Taylor 1991). Although both perspectives are important, I am here concerned with a third possibility, namely, the authenticity of desires. For the present purposes I take desires to be the most basic element in ordinary preference-forming and, thus, a basic element in decision-making. In brief, I hold that autonomy in medical settings mainly concerns decision-making, in the sense that bioethicists are interested in whether patients make autonomous healthcare decisions. Therefore, I phrase concerns of authenticity in terms of the authenticity of decisions, or more precisely in terms of the authenticity of desires.^1^

The notion of authenticity is relevant in several ways. A general theory of authenticity can be applied in common autonomy-protecting practices, such as, e.g., informed consent (cf. Eyal 2012). It is possible that such practices can be developed in light of insights from authenticity theory so that they better protect patient autonomy. But the notion is also relevant for paternalistic reasons. Sometimes, the principle of respect for autonomy is overridden by concerns for a patient’s well-being. Although compulsory care is rare, it is occasionally considered necessary. And, in some of those cases, the decision to put a patient in compulsory care is made with support from judgments of inauthenticity. That is, patients are sometimes subjected to compulsory care because they display what seems to be inauthentic desires (cf. Tan et al. 2006).

It must be noted that paternalistic interventions are not justified simply because a desire is found to be inauthentic. Paternalism requires support from independent moral arguments, such as the necessary degree of epistemic certainty of inauthenticity and the reasonable proportionality of the intervention. This article does not seek to provide practical guidance in those matters. It is beyond the scope of the present purposes to elaborate more precisely on the relationship between authenticity, autonomy, and paternalism. Here, the only concern is to determine what justifies judgments of inauthenticity.

Finally, I do not claim that my proposal is the only way to justify judgments of inauthenticity. The conclusion is not phrased in terms of necessary and sufficient conditions but should be understood as generally reason-giving in a larger framework of reflective equilibrium. Thus, applying it in practice requires substantial moral deliberation (cf. Beauchamp and Rauprich 2016).

### Two Taxonomies of Authenticity Theories

There have been two recent attempts at collecting theories of authenticity in taxonomies; Noggle (2005) and Ahlin (2018). Noggle’s taxonomy is important to the arguments in the next section as it distinguishes between so-called procedural and substantive theories of authenticity. The taxonomy I have proposed is important because it fleshes out two different kinds of theories that are conflated in Noggle’s taxonomy. As will be explained, these theories are fundamental to the arguments in the next section.

Noggle’s taxonomy builds on the observation that theories of authenticity begin with a base clause (2005, p. 88):

Element (or set of elements) E_1_ of the psychology of person S is authentic if…

Then, he orders different theories after which conditions they add to complete the clause. Three families of theories emerge (Noggle 2005, p. 88):

*Structural Condition Schema*: E_1_ is related in the right way to E_2_, where E_2_ is some other element (or group of elements) of S’s psychology.

*Historical Condition Schema*: E_1_ arose in the right way.

*Substantive Condition Schema*: E_1_ has the right content or causes S to believe, desire, intend, or do the right things.

Examples of “structural condition theories” include the notable autonomy theories proposed by Frankfurt (1971) and Dworkin (1988). According to those theories, a desire is authentic if the desire-holder identifies with it on a higher level of reflection. To illustrate, Noggle uses the example of an addict who has a first-order desire to use drugs, and a second-order desire to not use drugs: “When a person has both a first-order desire and a second-order desire not to have the first-order desire […] this repudiated first-order desire is properly regarded as ‘a force other than his own’” (p. 89). Thus, in those theories, a desire (element E_1_) is authentic if it complies with higher-level desires (E_2_). Structural condition theories have been supported in recent writings by, among others, Christman (2009), DeGrazia (2005), and Sjöstrand and Juth (2014).

In “historical condition theories,” desires are authentic if they have the right sort of causal history. “The motivating idea behind historical conditions seems to be that a psychological element is authentic if its history is free of the kinds of influences […] that seem to undermine authenticity” (Noggle 2005, pp. 93–94). Noggle refers to Dworkin, who offers examples of conditions that form the kind of influence that negates authenticity; “hypnotic suggestion, manipulation, coercive persuasion, subliminal influence, and so forth” (1988, p. 18).

Lastly, in contrast to the prior families of theories, “substantive condition theories” are not content-neutral. In substantive condition theories, the content of desires matter to the authenticity of the desires. The following is a hypothetical case which is sometimes used as an example to distinguish between substantive theories and content-neutral theories. Suppose that a woman lives with a man that regularly abuses her physically and verbally. The woman could choose to leave the man but chooses not to do so. Are the desires underlying woman’s choice not to leave the man authentic?

Content-neutral theories of authenticity are concerned with the processes of her choosing. They could conclude that the woman’s choice rests on authentic desires. In Noggle’s terminology, the woman’s decision-making processes could be structurally or historically conditioned so that there is no ground for concluding that her choice builds on inauthentic desires (although this conclusion is improbable).

By contrast, a substantive theory could reach the opposite conclusion, on the grounds that no matter how the woman’s decision-making processes are structurally or historically conditioned, the desire to stay with an abusing man cannot be authentic *because* it is the desire to stay with an abusing man; the desire has the wrong content. The reasons why the content is wrong vary between different substantive theories. For instance, one possible explanation is that one cannot authentically desire to fully submit oneself to the wishes of someone else. Submitting oneself fully to others’ wishes is to resign as a moral agent, which goes against the very idea of authenticity; one distinguishing factor between inauthenticity and authenticity is that the latter has to do with being self-driven in some sense. The woman cannot authentically choose to submit herself to the man, because one cannot authentically wish to be else-driven.

Content-neutral theories are commonly called “procedural.” Procedural theorists hold that a theory of authenticity should be content-neutral mainly because it should not be moralizing or enable undue paternalism. Essentially, it should be content-neutral because it should be morally neutral. Theorists from the substantivist tradition disagree, not least because of the reasons invoked above. The debate between theorists from the two traditions is ongoing (cf. Christman 2004; Oshana 2015), and while the distinction between procedural and substantive theories is relevant in the next section it may be left without further elaboration here.

The taxonomy I have proposed is not of authenticity theories, but of features that various theories share. In the taxonomy, different theories of authenticity are divided into three categories according to unique features. The categories are *sanctionism*, *originism*, and *coherentism* (Ahlin 2018, pp. 45–47).^2^ Here, by “sanctionist theories,” for instance, I intend a hypothetical theory which only displays sanctionist features, although the wording is only for pedagogical reasons; authenticity theories can display features from more than one category, and to different degrees. One strength of this taxonomy is that it shows that two distinct families of theories are conflated in Noggle’s taxonomy, namely, those that emphasize affirmative self-reflection and those that emphasize coherence. This is elaborated on below. One weakness is that the taxonomy only collects features shared by procedural theories of authenticity.

I call the distinguishing feature of sanctionist theories “affirmative self-reflection.” This feature is similar to the structural condition schema in Noggle’s taxonomy. Easily put, affirmative self-reflection is to critically scrutinize one’s own desires and approve of the result. For instance, suppose that a patient came to know precisely why she has the desire to refuse a medical intervention, reflected critically upon those causes, and concluded that she supports having the desire. The patient would have engaged in affirmative self-reflection and, according to sanctionist theories, her desire to refuse would be authentic.

The distinguishing feature of originist theories is very similar to the historical condition schema in Noggle’s taxonomy. In originist theories, desires are inauthentic if they have the wrong sort of origin. One example of an inauthentic desire is one which is “shaped by irrelevant causal factors, by a blind psychic causality operating ‘behind the back’ of the person” (Elster 1983, p. 16). Elster writes that “desires that have been deliberately chosen, acquired or modified—either by an act of will or by a process of character planning” are authentic (p. 21). That is, desires are authentic if they originate in the right kind of cognitive processes. On another originist account, desires are authentic if they originate in processes of self-discovery and self-definition (Ahlin 2018, p. 46; cf. Meyers 2001, 2005).

By introducing coherentism, the final category in my taxonomy, a distinguishing feature is fleshed out that is conflated in the structural condition schema in Noggle’s taxonomy. In coherentist theories, desires that deviate from the desire-holder’s full set of desires are inauthentic. For instance, in Christman’s theory of autonomy, a desire is authentic if it is not “alienated” upon self-reflection, “given one’s diachronic practical identity and one’s position in the world” (2009, p. 155). The notion of “practical identity” should here be understood to mean “a certain pattern of thinking and reacting which, generally speaking, is ours alone; it marks our character and personality” (p. 150). In short, a desire is inauthentic if it does not fit with how the desire-holder’s identity has developed over time, and how the identity is presently being sustained. Similarly, Miller argues that an action (here: desire) is inauthentic if it is “unusual or unexpected, relatively important in itself or its consequences, and [has] no apparent or proffered explanation” (1981, p. 24). Thus, in coherentist theories, desires are inauthentic if they are deviating.

## Judgments of Inauthenticity

### The Structure of the Argument

In this section, I spell out my proposal of what justifies judgments of inauthenticity. In short, the argument has three elements. The first element is a normative thesis determining under which conditions judgments of inauthenticity are justified. It is introduced in the next subsection. The second element is a set of indicators of inauthenticity, i.e., empirical factors that indicate whether the conditions in the first element are met. It is introduced in a subsequent section. The third element, which is also spelled out in an independent subsection, is a clause that delimits the scope of desires and desire-holders which may be justifiably subjected to judgments of inauthenticity. In a final subsection, the elements are collected and formulated as a proposal of what justifies judgments of inauthenticity.

I think of my arguments as contributing to theories in the sanctionist tradition. The tradition is the most influential, and any serious contribution to it should be of interest to autonomy theorists in general. However, I do not intend to defend sanctionism as such here; that is a different project. This also means that my arguments are intended to be neutral with regard to the content of desires, and thus only concern their procedural forms.

### The Dissenting Self-reflection Thesis

It has been pointed out that sanctionist theories suffer from epistemic problems that are difficult to overcome. Sjöstrand and Juth write (2014, p. 21):

For one thing, it is often difficult to come up with a full explanation as to why we have a certain desire, and even more difficult to make the necessary investigations in order to determine whether or not this explanation is correct.

It may be added that even if this problem is solved, it is also difficult to know whether affirmative self-reflection actually takes place (Ahlin 2018, p. 47):

[Observing a desire-holder’s endorsement of a desire] would require access to advanced (and currently unavailable) neuro-imaging technology, in addition to an in-depth knowledge of the psychological nature of endorsement. It would appear that sanctionism is, at the very least, impractical. [It] does not render observable and testable consequences without technology and scientific knowledge yet unheard of, if at all.

However, sanctionism remains a valid and strong theoretical ideal. Consider this thesis, which is formulated with sanctionism as a starting point:

**The dissenting self-reflection thesis:** Judgments of inauthenticity are justified if there is sufficient reason to believe that the desire-holder would disapprove of having the desire upon informed and critical self-reflection.

In it, affirmative self-reflection is re-stated as a negative. The dissenting self-reflection thesis does not claim to distinguish between authentic and inauthentic desires. The thesis states the conditions under which it is justified to judge that a desire is inauthentic, which means that there could be inauthentic desires that observers for some reason are not justified to call inauthentic. Although the thesis is sanctionist, it is more practical than the theoretical ideal. It facilitates the quest for empirical indicators of inauthenticity. From an observer’s epistemically inadequate point of view there are many reasons for a desire-holder to approve of her own desires, while reasons to disapprove of them are fewer, or at least easier to identify. That is, things that indicate inauthenticity are easier to observe than things that indicate authenticity. Therefore, re-stating affirmative self-reflection as a negative has at least one major epistemic merit.

It also has at least one moral merit. The dissenting self-reflection thesis includes a tacit assumption of authenticity; desires should be judged as authentic unless there is evidence of the opposite. There are moral reasons supporting this view. For instance, taking other people seriously, i.e., listening to what they say, respecting their wishes, treating them as “ends in themselves,” and so on, seems to require the assumption that they are acting from authentic desires. The dissenting self-reflection thesis complies with those reasons through its tacit assumption of authenticity. Therefore, there is at least one moral merit in building from the dissenting self-reflection thesis rather than from theses of affirmative self-reflection.

The aim of the arguments in what follows is to determine when there is reason to believe that a desire-holder would disapprove of having a desire upon informed and critical self-reflection. The aim is empirical. That is, the aim is to identify empirical factors that indicate that a desire is inauthentic. I have found two possible candidates in the recent literature on authenticity that, when combined, indicate inauthenticity. They are spelled out dialectically after this short but important subsection on a fixed point in the analysis.

### A Fixed Point in the Analysis

On the standard account of reflective equilibrium, the analysis allows for “fixed points” that are less sensible to re-evaluation than other matters included in the inquiry (Daniels 2016; Rawls 2001, pp. 29–30). For an illustration, the thesis that it is wrong to torture innocents for mere amusement can be held as a fixed point in an analysis of, for instance, the ethics of war. The present analysis holds one thesis as a fixed point, namely the following.

In one famous case, a 40-year old man developed a sexual interest in children that was later found to be causally connected to a brain tumor (Burns and Swerdlow 2003). When the tumor was removed the pedophilic symptoms disappeared. After some time, the man displayed the same symptoms again, and upon examination it was found that the brain tumor had returned. The causal connection between the man’s brain tumor and his sexual desires is clear beyond reasonable doubt. The man went through various medical procedures and a 12-step program for sexual addicts to be able to return to his family and his prepubescent stepdaughter, towards whom he had previously made subtle sexual advances.

Here, for reasons of stable and considered intuitions, I hold this thesis as a fixed point: *It is justified to treat the man’s sexual desires as inauthentic*. Holding the thesis as a fixed point does not entail any normative commitments. For instance, it does not mean that I do not hold the man accountable for his actions, or that I would support forced medical interventions aiming to remove the tumor or counteract its causal effects. I merely believe that few or no real cases are better suited to be held as fixed points in the present analysis; if it is not justified to treat the man’s sexual desires as inauthentic, it is perhaps never justified to treat any desire as inauthentic.

### Indicators of Inauthenticity

The originist (or “historical condition”) view that desires are authentic if they have the right sort of causal history is intuitively compelling. Desires that are “shaped by irrelevant causal factors” that operate “behind the back” of the desire-holder seem to be *prima facie* inauthentic. It is therefore an indicator of inauthenticity:

**The first indicator of inauthenticity:** It is a reason to believe that a desire-holder would disapprove of having a desire upon informed and critical self-reflection if it is known that the desire is due to causal factors that are not normal to how the desire-holder is otherwise construed, taking both physical and mental dispositions into consideration.

Applying it to the fixed point-case, the indicator provides a plausible explanation for why it is justified to treat the man’s sexual desires as inauthentic. However, it requires some elaboration.

Suppose, for the sake of argument, that the man had lived with the brain tumor and its causal influences from birth. He could then have felt a deep and stable connection between his personality and his sexual preferences (disregarding whether he found them morally acceptable) and developed a social identity and a way of life thereafter. He could, for instance, have thought of himself that, “I am the sort of person who cannot live close to playgrounds and schools,” and decided to live in solitude, cultivating an interest in botany, rock-climbing, or literature. In this case, the brain tumor is normal to how the man is otherwise construed. It would be less justified to believe that the man would disapprove of his desires upon informed and critical self-reflection. Perhaps it would not be justified at all, as the hypothetical man is known to feel a deep and stable connection between his personality and his sexual preferences. Therefore, the clause in the indicator stating that the causal factors must not be normal to how the desire-holder is otherwise construed is important. The clause also introduces a person-specific quality to judgments of inauthenticity, as what is normal to one person may not be normal to another (which the example in this paragraph illustrates).

However, the first indicator of inauthenticity does not have universal explanatory power. Consider, as a counterexample, a case in which a brain tumor causes a person to have desires that she already has. For instance, a sugar addict may have many reasons to love sugar. Perhaps her parents rewarded her with candy in her early childhood, thus “programming” her to have a certain cognitive attitude to sugar, and perhaps she lives next to a chocolate factory and cannot resist the always-present scent of sweets in her neighborhood. Suppose that this person develops a brain tumor causing her to have a desire for sugar. The tumor’s causal influences would be just one among many others, and it is therefore not obvious that the sugar addict would disapprove of having those desires upon informed and critical self-reflection. It should be concluded that although the first indicator is reason-giving, it does not by itself provide sufficient reason for judgments of inauthenticity to be justified.

The literature on authenticity includes other possible indicators of inauthenticity. Consider the coherentist view that a desire is inauthentic if it does not fit with the desire-holder’s practical identity. It renders a second indicator:

**The second indicator of inauthenticity:** It is a reason to believe that a desire-holder would disapprove of having a desire upon informed and critical self-reflection if it is known that the desire does not cohere with how the desire-holder’s identity has developed over time and is presently being sustained.

Applying the indicator to the fixed point-case, it provides an explanation for why it is justified to treat the man’s sexual desires as inauthentic. But, it needs to be elaborated.

People in general are rarely fully coherent beings. It is likely that most or all of us have conflicting desires. This is even more certain if we think of humans as intertemporal beings that exist over time; few people have fully coherent desire-sets over time from childhood to old age. Therefore, the second indicator should be understood as pointing at very serious deviations. A hypothetical person’s desire to drink beer for lunch and desire to be sober in the afternoon are conflicting, but the conflict is not serious enough for it to be justified to judge any of the two desires as inauthentic. But, in the fixed point-case, the man had deep sexual desires that were at odds with other deep desires, such as that of being part of a loving family and have a normal social life. His sexual desires negated life plans that were important to him. Therefore, they are serious enough to be a reason to believe that a desire is inauthentic.

However, as with the first indicator, the second indicator of inauthenticity lacks universal explanatory power. Consider, for instance, a person who does not suffer from a brain tumor, but who begins to act upon pedophilic desires because of the sudden realization that he has them. That is, suppose that the man’s sexual desires were not due to some non-normal causal factor, but that they were latent and emerged upon new stimuli. It would then not matter to judgments of inauthenticity that the desires conflict with the man’s practical identity. Therefore, as with the first indicator of inauthenticity, it should be concluded that although the second indicator is reason-giving it does not by itself provide sufficient reason for judgments of inauthenticity to be justified.

The two indicators seem to complement each other. Consider them in combination:

**A combination of the two indicators of inauthenticity:** There is reason to believe that a desire-holder would disapprove of having a desire to the extent that the desire is known to be due to causal factors that are not normal to how the desire-holder is otherwise construed, taking both physical and mental dispositions into consideration, and to the extent that the desire is known to be incoherent with how the desire-holder’s identity has developed over time and is presently being sustained.

The combination explains why it is justified to treat the man’s sexual desires as inauthentic in the fixed point-case, while withstanding the counterarguments that have been directed to the two indicators of inauthenticity as separate indicators. It avoids the argument directed to the first indicator regarding new factors that cause people to hold desires that they already have; because they are not deviating, they do not justify judgments of inauthenticity. It also avoids the argument directed to the second indicator regarding normal factors causing incoherent desires; because the factors are not of a certain kind, they do not justify judgments of inauthenticity. Thus, the combination seems to provide a reasonable explanation while withstanding criticism that refute the two indicators of inauthenticity as separate indicators.

However, there are forceful counterexamples also to the combination of the two indicators. Suppose that the man in the fixed point-case already was a pedophile, but that the brain tumor caused his sexual desires in children to disappear. The causal factors would not be normal to how he is otherwise construed, and his new set of desires would be seriously incoherent with his practical identity, yet it seems counterintuitive to conclude that it is justified to believe that the man would disapprove of his new desire-set upon informed and critical self-reflection. On the contrary, he might view the brain tumor as a blessing.

This counterexample is different from the previous; it targets the content of the desires rather than their procedural forms and thus comes from what in Noggle’s terminology is called a “substantive condition schema.” And, it seems to succeed in one aspect; the combination of the two indicators only seems to justify judgments of inauthenticity when the desires under scrutiny are bad, in some sense. When the desires are good, in some sense, the combination provides counterintuitive conclusions. This can be further illustrated.

In his popular book *The Man Who Mistook His Wife for a Hat*, the neurologist Oliver Sacks reports of a 90-year-old woman who had noticed a “change” (1985, Ch. 11). Around her 88^th^ birthday, she had begun to feel energetic, alive, younger; she had always been shy, but now she flirted with young men, made jokes, and had fun. Her friends thought that her frisky behavior was inappropriate at her age. The woman was feeling extremely well—*too* well—and realized that it could be “Cupid’s disease.” She had received treatment in her youth, but the infection had only been suppressed, not eradicated. The woman was right, she had neurosyphilis. Upon confirmation of her hypothesis, the woman stated that she did not want to be cured from the infection, as she enjoyed its positive effects, but that she did not want it to get worse either.^3^ Contrary to what the combination of the two indicators would suggest, the woman did not disapprove of her desires upon informed and critical self-reflection, in spite of the fact that they were both alien and deviating.

Thus, the combination seems to justify judgments of inauthenticity when the desires under scrutiny are bad in some sense, but not when they are good in some sense.^4^ This observation needs to be sorted out and answered.

### A Delimiting Clause

The dialectic in the above subsection builds on theories and propositions from the procedural tradition of autonomy theorizing. Yet, the final counterexample which convincingly refutes the combination of the two indicators of inauthenticity seems to be substantive. The example succeeds in showing that the combination only renders plausible conclusions regarding desires with a certain kind of content. Thus, the dialectic appears to rest on tacit substantive assumptions that must be made explicit and explained.

In the fixed point-case, the assumption is that it would be better for the man to not have those sexual desires. It would be better according to objective moral standards, as not being a pedophile is morally better than being one. The clause could be added to the combination of the two indicators, “unless the desire is better according to objective moral standards.” Judgments of when there is sufficient reason to believe that a desire-holder would disapprove of a desire would then be normative. Such moralizations are precisely what proceduralists wish to avoid. The substantive assumption that must be made explicit can therefore not be objective in the sense reflected by this line of thought.

It would also be better for the man to not have those sexual desires according to his own subjective moral standards. It can reasonably be assumed that the man did not want to have those desires, as he underwent a 12-step program for sexual addicts in addition to the various medical procedures to be able to return to his family. A different clause could thus be added to the combination, “unless the desire is better according to subjective moral standards.” However, judgments of inauthenticity would then be self-referential and non-guiding. The full theory would essentially carry the meaning, “there is reason to believe that the desire-holder would disapprove of having the desire if it is new and deviating, unless the desire-holder approves of having it,” or simply, “the desire-holder would disapprove of having the desire unless she approves of having it.” Therefore, the substantive assumption that must be made explicit cannot be subjective in the sense reflected by this line of thought either.

However, there is one possible middle-way between those two lines of thought. The following delimiting clause can be added:

**Delimiting clause:** Here, judgments of inauthenticity only target desire-holders who are known to carry a general wish to live according to the prevailing social and moral standards, and desires that are seriously undesirable according to those standards.

The man in the fixed point-case is such a desire-holder, which is known from his efforts to defeat the symptoms of his brain tumor, and his desires are undesirable accordingly, which is known from observations of the prevailing social and moral standards. More fully spelled out, the judgment is then that there is reason to believe that the man in the fixed point-case would disapprove of having his desires as they are alien, incoherent, and undesirable according to the standards which it is known that he wishes to follow. The delimiting clause also goes well with the old woman whose syphilis caused her to have alien and deviating desires. Her frisky mood and behavior is not undesirable enough either socially or morally. Therefore, the woman’s desires are not of the kind that may justifiably be targeted by judgments of inauthenticity.

I expect four immediate objections to the delimiting clause. Each is due to the fact that it brings normative content into judgments of inauthenticity.

First, proceduralist protests can be expected. Proceduralists hold that judgments of inauthenticity should be content-neutral because they should be morally neutral. However, they should not worry. There is no risk for undue paternalism, as the clause also states that judgments of inauthenticity here only target people who are known to wish to live according to the same normative content that the judgment builds on. And, the normative content does not concern the distinction between authentic and inauthentic desires, but when it is justified to treat desires as inauthentic, i.e., an inherently normative problem. There must be moralization, and proceduralists should grant that applying the desire-holder’s own moral standards is at least less problematic than applying someone else’s.

Second, it may be objected that adding the delimiting clause to the theory entails that judgments of inauthenticity would be self-referential and non-guiding, in the sense discussed above. The full theory would essentially carry the meaning, “the desire-holder would disapprove of having the desire unless she approves of having it.” The objection obscures an important difference between the two additions. In the above discussion, the worry of circularity is due to the fact that the addition to the combination reads, “unless the desire is better according to subjective moral standards.” In that addition, the desire-holder’s attitude to the specific desire which is under scrutiny is known and added to the analysis. In the delimiting clause, on the contrary, nothing is said about any specific desire. Instead, the delimiting clause adds the desire-holder’s attitude toward the prevailing social and moral standards (according to which specific desires may be good or bad). Thereby, the addition is substantive enough to be analytically potent, while being sufficiently content-neutral to avoid being problematically circular.

Third, substantivist protests can also be expected. Substantivists hold that the content of a desire should be of a certain kind for the desire to be authentic, and the normative content which is here brought into judgments of inauthenticity is not objective. If the prevailing moral standards are wicked, that wickedness is brought into the judgment and is thus acted upon—surely, the substantivist might say, it must be wrong in and by itself to act upon wicked moral standards. However, the task is here to justify judgments of inauthenticity in light of the desire-holder’s values. In all other matters, it is a value-neutral project. The current project aims to contribute to the justification of judgments of the possible inauthenticity of desire-holders’ wicked views, not the justification of their views as such.

Fourth, delimiting the analytical scope to desire-holders who are known to wish to live according to the prevailing social and moral standards may entail that a lot of people are left out. The current analysis does not provide guidance with regard to people whose values and motivational sets are unknown or deviating from the prevailing social and moral standards. My answer is simply that including those people is not morally justified, precisely because their values and motivational sets are unknown or deviating. The present theory ultimately aims to be proceduralist and thus both non-moralizing and anti-paternalist. Therefore, it is not a problem that the analytical scope is narrow in this sense. On the contrary, this narrowness counts in favor of the theory as a whole.

### Justifying Judgments of Inauthenticity

Adding the delimiting clause to the dissenting self-reflection thesis and to the combination of the two indicators of inauthenticity results in the following:

1. Here, judgments of inauthenticity only target desire-holders who are known to carry a general wish to live according to the prevailing social and moral standards, and desires that are seriously undesirable according to those standards.
2. Judgments of inauthenticity are justified if there is sufficient reason to believe that the desire-holder would disapprove of having the desire upon informed and critical self-reflection.
3. There is reason to believe that a desire-holder would disapprove of having a desire to the extent that it is known to be due to causal factors that are not normal to how the desire-holder is otherwise construed, taking both physical and mental dispositions into consideration, and to the extent that it is known to be incoherent with how the desire-holder’s identity has developed over time and is presently being sustained.

1 through 3 justifies judgments of inauthenticity. In shorter terms, and in light of the arguments above, the justification may read:

For persons who wish to live according to the prevailing social and moral standards and desires that are seriously undesirable according to those standards, it is justified to judge that a desire is inauthentic to the extent that it is due to causal factors that are alien to the person and to the extent that it deviates from the person’s practical identity.

## Concluding Remarks

To conclude, it is not clear how the notion of authenticity should be understood, nor what its place is in the contemporary autonomy-oriented bioethical paradigm. However, it is both common and reasonable to treat the notion as concerning desires. As such, it is a problem to determine what justifies judgments of inauthenticity. A content-neutral solution to that problem has been proposed building from the sanctionist tradition (a starting point and a tradition which both require elaborate and independent defenses).

I do not claim that my proposal is the only way to justify judgments of inauthenticity. Instead, it should be understood as generally reason-giving in a larger framework of reflective equilibrium, and applying it requires substantial moral deliberation. For instance, very little has been said about what it means that a desire is “seriously” deviating from a person’s practical identity. Further guidance in that particular matter is found in, e.g., Christman (2009, pp. 149–156). Likewise, 1 through 3 and the summarized proposal are expressed in terms of degrees rather than in necessary or sufficient conditions. Therefore, it must always be a matter of deliberation to determine, e.g., to which extent a desire is known to be alien to the desire-holder. Further guidance in how to apply the proposal in practice may be found in the methodological discussions in Beauchamp and Childress (2013) and Beauchamp and Rauprich (2016).

Furthermore, it must be noted that decisions are not autonomous only because it is not justified to judge that the underlying desires are inauthentic. Although the desires must be treated as authentic, the desire-holder may, e.g., be incapable of realizing and assessing the implications of the decision. In short, authentic decisions do not necessarily amount to autonomous decisions.

I have here attempted to bridge the gap between theoretical ideals of authenticity and real authenticity-related problems in practical biomedical settings. Future contributions to authenticity theory may determine whether I have succeeded in that aim.

## Footnotes

1 Also, the focus on desires is common in the theoretical tradition which I aim to contribute to; see e.g. Noggle (2005), Sjöstrand and Juth (2014), and Taylor (2005b).

2 Each category can be divided in two classes, namely *cognitivism* and *non*-*cognitivism*, although these will be left out of the present analysis.

3 Sacks eventually found a treatment that conformed to the woman’s wishes.

4 One plausible line of thought is instead that judgments of inauthenticity may be justified in either case, but that they are only interesting when the desire under scrutiny is bad in some sense. However, mainly for reasons of space, this possibility is not further explored here.
